# Combining and comparing EEG, peripheral physiology and eye-related measures for the assessment of mental workload

**DOI:** 10.3389/fnins.2014.00322

**Published:** 2014-10-14

**Authors:** Maarten A. Hogervorst, Anne-Marie Brouwer, Jan B. F. van Erp

**Affiliations:** TNO Human Factors, Netherlands Organisation for Applied Scientific ResearchSoesterberg, Netherlands

**Keywords:** EEG, physiology, eye, workload, classification, combination, ECG, skin conductance

## Abstract

While studies exist that compare different physiological variables with respect to their association with mental workload, it is still largely unclear which variables supply the best information about momentary workload of an individual and what is the benefit of combining them. We investigated workload using the n-back task, controlling for body movements and visual input. We recorded EEG, skin conductance, respiration, ECG, pupil size and eye blinks of 14 subjects. Various variables were extracted from these recordings and used as features in individually tuned classification models. Online classification was simulated by using the first part of the data as training set and the last part of the data for testing the models. The results indicate that EEG performs best, followed by eye related measures and peripheral physiology. Combining variables from different sensors did not significantly improve workload assessment over the best performing sensor alone. Best classification accuracy, a little over 90%, was reached for distinguishing between high and low workload on the basis of 2 min segments of EEG and eye related variables. A similar and not significantly different performance of 86% was reached using only EEG from single electrode location Pz.

## Introduction

In the literature, mental workload has been associated with a range of physiological variables. These include heart rate (e.g., studies as reviewed by Vogt et al., 2006), different types of heart rate variability (reviewed by Hancock et al., 1985; Aasman et al., 1987), pupil size (reviewed by Beatty, 1982; May et al., 1990; Porter et al., 2007; Hampson et al., 2010), eye blink frequency and duration (Wilson and Fisher, 1991; Brookings et al., 1996; Veltman and Gaillard, 1996, 1998), electrodermal measures (Kohlisch and Schaefer, 1996; Reimer and Mehler, 2011), respiration frequency (Wientjes, 1992; Mehler et al., 2009; Karavidas et al., 2010) and various variables derived from EEG (most prominently power in the alpha and theta band—reviewed by Brouwer et al. (2012).

A question that arises when one aims to put this knowledge into practical use is which variable(s) one should measure in order to get the best workload assessment for a specific individual. It is not easy to answer this question based on the current literature because of several complications. Firstly, only a limited set of variables is recorded and analyzed in each study, precluding easy comparison of performance across variables. Secondly, variables are often analyzed and reported at a group level rather than used to assess workload in an individual. Associations between physiological variables and workload as found using a group level analysis may not generalize to the case of assessing workload in an individual since they may not be sufficiently strong to reliably assess workload at a certain moment in time for a single individual. On the other hand, physiological responses to workload may be consistent within and not between individuals, which would result in variables that are seemingly non-responsive to workload at a group level while they are actually valuable for assessing workload on an individual basis. Finally, many workload studies suffer from experimental flaws in which workload levels are confounded with for instance body movements (potentially affecting heart rate and related variables) or visual information processing (potentially affecting eye- and EEG based variables). We here aim to provide an overview of the workload assessment performance of a rather broad range of variables within the context of an experiment in which visual input and the amount of body movements are constant across workload levels. Classification analyses are used to get an impression of the quality of workload estimation within an individual. While analyses are performed offline, we simulate an online^1^ situation, where our classification models are trained on data acquired at the start of the experiment and tested on data acquired at the end of the experiment, therewith avoiding inflation of classification accuracy due to time dependencies. The same data have been analyzed on a group level in Brouwer et al. (2014). That study gives an overview of the general magnitude and direction of effects of the different conditions on the studied variables.

Besides examining how well different variables can be used to estimate workload on their own, we examine to what extent combination of different variables improves performance. As discussed later, while some studies seem to suggest that assessment of mental state improves when combining physiological variables, reported improvements often are modest and not statistically significant or not statistically tested. We examine different ways of combining variables. Below we review the literature and formulate hypotheses as to what we expect to find.

### Sensitivity of single variables to workload

Studies on (neuro) physiological correlates of workload (or mental load) go back to at least the early sixties (Kalsbeek and Ettema, 1963). A range of variables has been examined over the years such as heart rate, different types of heart rate variability, pupil size, eye blink frequency and duration, saccade and fixation related measures, electrodermal measures, respiration, blood pressure, chemical measures, EMG and neurophysiological variables derived from EEG. To our knowledge, a substantial, recent review of physiological responses to workload is lacking. There does not seem to be an obvious “winning” variable that can effectively be used to determine workload. One review study (Hancock et al., 1985) suggested heart rate variability as the most reliable measure, whereas another (Vogt et al., 2006) reviewed 19 studies in which heart rate variability was not even recorded. In these studies, heart rate seemed to be relatively reliable. Most studies that examined EEG spectral variables next to physiological variables such as different eye and heart related measures, concluded or suggested EEG to be the most sensitive or promising indicator of workload (Brookings et al., 1996; Taylor et al., 2010; Christensen et al., 2012). The study by Christensen et al. (2012) showed that classification accuracy using only EEG data was only marginally lower compared to adding information about heart rate, blink rate, blink amplitude, blink duration and EOG. Berka et al. (2007) argue in their introduction that EEG is the only physiological signal that has been shown to accurately reflect subtle shifts in workload. However, in the three studies favoring EEG just mentioned, as well as in many other workload studies, workload was manipulated in the context of simulated realistic tasks involving potential confounds such as speech, body movement and visual information. In a recent study (Brouwer et al., 2014) we examined effects of workload and time using a task that controls for these kinds of confounds. Repeated measures ANOVA analyses did not mark EEG as the source of information that “best” indicated workload. Highly significant effects of workload were found for EEG in the alpha frequency band but also for mean and minimum skin conductance level, respiration frequency, heart rate, high frequency heart rate variability and pupil size. No significant effects were found for EEG in the theta frequency band, mid frequency heart rate variability, number of blinks and blink duration. Still, this study does not indicate which variables would be most useful for assessing workload based on a limited amount of physiological data of a single individual. This is especially the case since Brouwer et al. (2014) highlighted (strong) effects of time on most of the measured variables which could potentially complicate their use.

### Combining variables - processes underlying the association between workload and physiological variables

Being interested in combining physiological variables in order to arrive at a better assessment of workload, it is of special importance to examine the background of the association between the variables and workload. This is because using a combination of variables reflecting workload is especially expected to improve workload assessment if these variables are not all associated with the same but rather with different aspects of workload. As described below, high workload likely goes hand in hand with increased cognitive processing, increased (emotional) arousal and increased energy demand; aspects of workload that are presumably reflected by different physiological variables that have all been associated with workload before.

### Cognitive processing—EEG

EEG alpha activity (power in the 8–12 Hz band) has been linked to idling (Pfurtscheller et al., 1996), default mode brain activity (Laufs et al., 2003; Jann et al., 2009) and cortical inhibition (Foxe et al., 1998; van Dijk et al., 2008; Brouwer et al., 2009). This suggests that this measure would reflect different levels of workload, with high alpha for low levels of workload which indeed was reported in several workload studies (e.g., Fink et al., 2005; Brouwer et al., 2012). Another EEG frequency band that has been related to workload associated processes is theta (4–8 Hz). Evidence for an association between theta and working memory processes or mental effort has been summarized in several reviews by Klimesch (1996, 1997, 1999). Theta increases as task requirements increase (e.g., Miyata et al., 1990; Raghavachari et al., 2001; Jensen and Tesche, 2002; Esposito et al., 2009). A number of studies on workload reported both alpha and theta effects (e.g., Gundel and Wilson, 1992; Brookings et al., 1996; Gevins et al., 1998; Fournier et al., 1999).

Not only EEG spectral variables, also Event-Related-Potentials (ERPs) have been found to reflect different levels of workload. The P300 component of the ERP is a peak occurring 300 ms or somewhat later after an attended stimulus has been presented. It is thought to reflect attentional and working memory processes (Polich and Kok, 1995; Polich, 2007) and it is in particular this component that has been reported to decrease with increasing levels of memory or workload (Watter et al., 2001; Kida et al., 2004; Raabe et al., 2005; Allison and Polich, 2008; Evans et al., 2011; Pratt et al., 2011). Besides the P300, earlier ERP components like the N100 (Kramer et al., 1995; Ullsperger et al., 2001; Allison and Polich, 2008) the N200 (Kramer et al., 1995), the P1 (Pratt et al., 2011) and a positive-negative component between 140 and 280 ms (Missonnier et al., 2003, 2004) have been found to respond to task difficulty or workload. Finally, late positive or negative slow waves have been related to high memory load (Ruchkin et al., 1990) and amount of resource allocation (Rösler et al., 1997).

### Arousal and energy demand—peripheral physiology

High mental workload is associated with high mental effort (Hockey, 1986; Gaillard and Wientjes, 1994). Mental workload or mental effort is associated with a decrease of the parasympathetic (“rest or digest”) autonomous nervous system activity and an increase in sympathetic (“fight or flight”) activity (Mulder and Mulder, 1987; Gawron et al., 1989). These changes in autonomous nervous system activity can be estimated through several peripheral physiological measures such as skin conductance (Roth, 1983), heart rate and heart rate variability (Berntson et al., 1997).

Electrical skin conductance varies with the moisture level of the skin. Since the sweat glands are controlled by the sympathetic part of the autonomous nervous system (Roth, 1983), electrodermal measures indicate the level of sympathetic activity or arousal. A large body of literature describes the positive effect of arousal on skin conductance (e.g., Winton et al., 1984; Greenwald et al., 1989; Boucsein, 1992, 1999; Brouwer et al., 2013). While increases in skin conductance may be viewed as reflecting sympathetic activity as a consequence of arousal due to mental effort, Reimer and Mehler (2011) and Kohlisch and Schaefer (1996) interpret their findings of heightened skin conductance with increased workload as reflecting emotional arousal.

Heart rate and its variability are affected by activation and suppression of both the sympathetic and parasympathetic nervous systems (Berntson et al., 1997). At normal breathing frequencies, fast changes in heart rate (0.15–0.50 Hz) reflect the adjustment of heart rate to breathing: breathing causes changes in blood pressure and by adapting heart rate, blood pressure is kept around a certain point (Mulder, 1980; Aasman et al., 1987). Also, the adaptation to breathing facilitates gas exchange between the lungs and the blood (Grossman and Taylor, 2007). High frequency heart rate variability reflects only the (fast) parasympathetic nervous system (Berntson et al., 1997). Mental effort has been reported to have the largest effect upon the mid-band (0.07–0.14 Hz; Mulder, 1980; Aasman et al., 1987). This band reflects not only parasympathetic but also sympathetic activity (Berntson et al., 1997; Veltman and Gaillard, 1998). For both bands, suppression of parasympathetic activity (associated with high workload) results in lower adaptation to changes in blood pressure and hence less heart rate variability.

Mental workload being associated with increased arousal and neural activity increases metabolic demand, which is probably the cause of observed increases in heart rate and respiration frequency with workload (Veltman and Gaillard, 1998).

### Eye-related measures

Pupil dilation is not only caused by decreasing luminance but also by increasing workload (Beatty, 1982; May et al., 1990; Porter et al., 2007; Hampson et al., 2010). Consistent with this, the frontal cortex is involved in controlling pupil dilation (Hampson et al., 2010). The underlying function is unclear, but the fact that the effect has been observed in studies that varied task difficulty without varying the visual environment (Kahneman and Beatty, 1966; Kahneman et al., 1969) indicates that it does not primarily serve purposes related to visual perception. Reduction of blink frequency and duration with workload could be attributed to maximizing detection of visual information (Bauer et al., 1987; Fogarty and Stern, 1989). In this sense, the sensitivity of these parameters can often be explained by high workload being confounded by the presence of much visual information.

### Three sensor groups

In sum, we can loosely divide physiological variables found to be associated with workload into three, what we call “sensor groups” that are assumed to reflect different aspects of workload. EEG measures are expected to mainly reflect cognitive processes. Peripheral physiological measures reflect arousal and energy demand. The third group of eye related measures have probably partly been found to covary with workload due to the often occurring confound of the amount of visual information, but for pupil dilation, the reason for its association with workload is unclear. Considering the idea that they reflect different aspects of workload, combination of these groups is expected to lead to better classification accuracy than either group alone, especially for the combination of EEG and peripheral physiology.

### Combining variables—fusion techniques

In previous workload studies, EEG has been combined with other physiological signals for assessing workload. Coffey et al. (2012) found that classification of workload based on EEG was more accurate than when based on fNIRS (functional Near Infrared Spectroscopy), and that combining the two did not increase classification performance. Wilson and Russell (2003, 2007) combine respiration (Wilson and Russell, 2003), EEG, EOG and heart rate in their classification models to assess workload in simulated aviation-related tasks. However, they do not report on the relative contribution of these different signals to classification performance. Christensen et al. (2012) assessed workload in simulated remote piloting. Their classification models were based on EEG, EOG, heart rate, blink rate, blink amplitude, and blink duration. They did not extensively report on the relative contribution of these variables, but mention that when classification was performed on the basis of EEG only, classification accuracy hardly decreased (about 2%). Chanel et al. (2006) studied the relative contribution of EEG and peripheral physiological signals (skin conductance, heart rate, blood pressure, respiration and temperature) on classifying mental states as elicited by emotional pictures. They also did not find a strong advantage of fusion of EEG and physiology over EEG alone.

In all of these studies, combination of variables from different domains was achieved by simple concatenation of the input feature vectors. However, when combining EEG data with physiology, the large difference in length of the feature vectors forms a potential problem. While EEG spectral features are captured by power values in different frequency bands at different electrodes amounting to a large number of features, physiological or eye related features such as pupil size and heart rate are typically each represented by just one (average) value. This could lead to a priori small added value of these features. A possible solution is to use higher order combination of information by combing the assessments based on the various types of features. Such a method was used by Chanel et al. (2009) who studied classification of different emotions as elicited by emotional recall. Classification decisions were made by two different EEG based classifiers and one classifier based on physiology (skin conductance, heart rate, blood pressure and respiration) and these decisions were then combined. Adding the worst performing EEG set to the best performing one increased classification accuracy (that was generally between 70 and 80% for a two class problem) with about 2–4%, and adding physiology on top of that resulted in an additional increase of up to almost 3%. There was no direct comparison with the concatenation method (using all features as input to a single classifier) though the authors mention that this method did not lead to an increase in accuracy.

The improvements in classification accuracy as found by Chanel et al. (2009) are relatively small and are probably not statistically significant. However, the trend is positive and we think it is worthwhile to examine the case for workload where EEG and other types of signals are expected to complement each other. We will compare the discussed ways of combining information, i.e., fusion at the feature level or at the decision level. When combining decisions from different classifiers, a confidence measure of the decision is useful. Such a confidence measure is given by an elastic net model with logistic regression (Friedman et al., 2010). Therefore, besides using linear Support Vector Machine as the more standard classification model, we also use an elastic net model. This enables us to weigh information of the different sources before averaging (a similar method was used by Chanel et al., 2009). The potential advantage of fusion at a decision level is that smaller feature vectors reflecting physiology or eye related measures do not run the risk to be “flooded” by EEG—the disadvantage of fusion at the decision level is that interactions between different features or feature sets may be missed.

### Current study: overview and hypotheses

We study workload in an experiment in which we control for visual input and the amount of body movements by using an n-back task to vary workload. This task requires participants to indicate of each of successively presented letters whether it is a target or not. Workload is low when the target letter is an “x” (0-back), intermediate when the target letter is the same as the one before (1-back) and high when the target letter is the same as two letters before (2-back). In this task, visual input and number of button presses are the same across workload levels. This means that effects of workload can really be attributed to differences in mental processes and cannot be due to different amounts of hand or eye movements in the high workload condition compared to the low workload condition.

We determine the value of individual features and combinations for the assessment of individual workload level using individually trained classification models. We simulate an on-line situation in which a model is tuned to an individual using data from the first part of the experiment and in which the workload is predicted for the last part of the experiment. We record EEG, skin conductance, respiration, ECG, pupil size and eye blinks. Various variables are extracted from these measurements and used as features in classification models. Firstly, we examine how well classification models based on the various individual features perform. We expect EEG features to perform best given indications from earlier studies and given the fact that EEG is expected to reflect what can be considered to be the core of mental workload, namely cognitive processing. Next, we will look at combinations of features. We start by combining features originating from the same sensor (e.g., heart rate and heart rate variability that can both be determined from ECG). While these are not expected to strongly improve classification performance since they are probably largely reflecting the same underlying process, we think it is worth trying for the practical reason that these features are available without additional costs (i.e., without having to use an additional sensor). Subsequently, we combine features from different sensor groups “EEG,” “Physiology,” and “Eyes.” Especially the combination of EEG with Physiology is expected to improve classification performance since these groups are assumed to reflect different general physiological processes associated with workload. For analyses at the sensor group level, we check whether taking time into account improves classification performance. An improvement of including information about time of measurement may be expected based on finding general effects of time on physiological variables (e.g., Fairclough et al., 2005; Brouwer et al., 2014). For analyses at the sensor group level, we also compare fusion at the feature level to fusion at the decision level. We use both SVM and elastic net classification models.

## Materials and methods

### Participants

Data of 14 participants are analyzed in this study. Participants were aged between 23 and 40 years (mean age 27.9), 8 female and 6 male. The experiment was performed in accordance with the local ethics guidelines and participants gave written informed consent^2^.

### Materials

Stimuli (letters), subjective workload scales and announcements about the type of the n-back task to follow were presented on a Tobii T60 Eye Tracker monitor, at a distance of about 50 cm from the participants' eyes. Feedback about task performance was presented through Labtec LCS-1050 speakers in the form of beeps. Participants used a keyboard to indicate whether presented letters were targets or non-targets. Which of the keys (1 or 2 on the numerical pad) indicated “target” and which “non-target” was counterbalanced between participants. Participants used the mouse to rate subjective workload on a scale (RSME) between the stimulus blocks.

EEG (electro encephalogram) was recorded through a g.tec USBamp and g.tec Au electrodes placed at Fz, FCz, Pz, C3, C4, F3, and F4, referenced to linked mastoid electrodes. A ground electrode was placed at FPz. Impedance was kept below 5 kΩ. EEG data were filtered by a 0.1 Hz high pass- and a 100 Hz low pass filter and sampled with a frequency of 256 Hz (USB Biosignal Amplifier, g.tecmedical engineering GmbH).

ECG (electro cardiogram) and skin conductance were recorded using a MindWare BioNex 8-slot chassis with a 3-channel Bio-Potential and GSR amplifier. A 4-channel transducer amplifier was used to measure respiration. For ECG measurement, self-adhesive 1 1/2″ electrodes with 7% chloride wet gel were attached just below the right collarbone, just below the left lower rib and above the right hip. To record skin conductance, two self-adhesive 1 5/8″ electrodes with 1% chloride wet gel were attached to the palm of the left hand that was not used for pressing the keys—one below the thumb and one below the little finger. Respiration was recorded using an elastic band around the waist at the height of the lower side of the sternum. MindWare's BioLab software was used to acquire ECG, skin conductance and respiration. These signals were sampled with a frequency of 300 Hz. They were acquired with a gain setting of 1000, 10, and 500 and filtered with a 0.5, 1, and 5 Hz high-pass filters, respectively.

Pupil size, blink rate and blink duration were measured using a Tobii T60 Eye Tracker that was integrated into an 17″ monitor. Recording frequency was 60 Hz. All signals were synchronized using the TCAP signal from The Observer XT (Zimmerman et al., 2009).

We used the RSME scale (Rating Scale Mental Effort, Zijlstra, 1993) to measure subjectively experienced mental effort. This scale runs from 0 to 150 with higher values reflecting higher workload. It has nine descriptors along the axis, e.g., “not effortful” at value 2 and “rather effortful” at value 58. Verwey and Veltman (1996) concluded this simple one-dimensional scale to be more sensitive than the often-used NASA-TLX (Hart and Staveland, 1988).

### Task

Participants viewed letters, successively presented on a screen. For each letter, they pressed a button to indicate whether the letter was a target or a non-target. In the 0-back condition, the letter x is the target. In the 1-back condition, a letter is a target when it is the same as the one before. In the 2-back condition, a letter is a target when it is the same as two letters before. With this version of the n-back task, the level of workload is varied without varying visual input or frequency and type of motor output (button presses). A 3-back condition was not used, due to evidence that many participants find it too difficult and tend to give up (Ayaz et al., 2007; Izzetoglu et al., 2007).

Participants were informed after every button press whether it was a correct decision by a high (correct) or a low (incorrect) pitched tone. This was intended to help the participant, who in our experiment switched rather often between n-back conditions, and to increase the likelihood that participants would decide to invest effort since the participant knew the experiment leader would hear the sounds as well.

### Stimuli

The letters used in the n-back task were black (font style: Matlab standard, approximately 3 cm high) and were presented on a light gray background. The letters were presented for 500 ms followed by a 2000-ms inter-stimulus interval during which the letter was replaced by a fixation cross. In all conditions, 33% of letters were targets. Except for the letter x in the 0-back task, letters were randomly selected from English consonants. Vowels were excluded to reduce the likeliness of participants developing chunking strategies which reduce mental effort, as suggested in Grimes et al. (2008).

### Design

The three conditions (0-back, 1-back, 2-back) were presented in 2-min blocks divided across four sessions. Each session consisted of two repetitions of each of the three blocks. Thus, for each of the three conditions participants performed 4 sessions ^*^2 repetitions = 8 blocks. In each block, 48 letters were presented, 16 of which were targets. The blocks were presented in pseudorandom order, such that each condition was presented once in the first half of the session and once in the second half of the session, and that blocks of the same condition never occurred directly after each other. Before each session was a baseline block of 2 min in which the participant quietly fixated a cross on the screen. With 4 sessions ^*^2 repetitions ^*^3 conditions, plus 4 sessions ^*^1 baseline block, the total duration of the n-back task was 56 min.

### Procedure

After entering the lab, participants read and were explained about the experimental procedure. They then signed an informed consent form. The physiological sensors were attached and the Tobii eye tracker was calibrated. The three conditions were practiced up to the point that the participant was familiar with the task. Regardless of this, all participants completed at least one block of the 2-back task in order to also practice the RSME rating that appeared at the end of the block. It was stressed that the 2-back task could be difficult, but that even when the participant thought it was too difficult he or she should keep trying to do as well as possible. Participants were asked to avoid movement as much as possible while performing the task and to use the breaks in between the blocks to make necessary movements. Before the start of each block, the participant was informed about the nature of the block (rest, 0-back, 1-back, or 2-back) via the monitor. After each block, the RSME scale was presented and the participant rated subjective mental effort by clicking the appropriate location on the scale using the mouse. The next block started after the participant indicated to be ready by pressing a button. Between sessions, participants had longer breaks, chatting with the experiment leader or having a drink.

### Definition of features

EEG data were filtered by a 0.1 Hz high pass- and a 100 Hz low pass filter and sampled with a frequency of 256 Hz (USB Biosignal Amplifier, g.tecmedical engineering GmbH). Afterwards data was processed and analyzed using Matlab and the FieldTrip open source Matlab toolbox (Oostenveld et al., 2011). Epochs starting at 500 ms before stimulus onset and ending 2000 ms after were shifted such that the mean of the first 500 ms was zero. No eye blink artifacts were removed before classification which makes the implementation of online classification easier. Our previous analysis (Brouwer et al., 2012) showed that with EOG performance was not better or contribute to EEG-based workload classification, indicating that performance is only expected to get better when removing them. Over each block and each of the 7 EEG-channels (C3, F3, C4, F4, Fz, Pz, FCz) we calculated the average ERP over all trials after resampling the data to 100 Hz. The (*N* = 101) samples between 0 to 1 s as ERP-features. Similarly, for each of the trials and channels the spectral power over complete trials (from −0.5 to +2.0 s) was calculated in (*N* = 37) bands ranging from 2 to 20 Hz (in steps of 0.5 Hz) following an FFT approach using a single Hanning taper. Next, the average spectral power was determined for each block and channel (by averaging over all trials within a block). Trials with extreme variance in the signal as defined by a standard deviation above 100 μV were discarded before calculating the average ERP and spectral power features (1% of the data). Apart from using the “raw” ERP and power spectra of the various EEG-channels we also used alpha power and theta power as feature input for classification. As a measure of alpha power we used the average over the natural log transformed power within the frequency band ranging from 8 to 13 Hz. As a measure of theta power we used the average over the natural log transformed power of frequencies between 4 and 8 Hz. Models that included alpha power and/or theta power as features did not also include the raw power values. Additionally, we examined alpha power of EEG as only recorded at Pz, theta power as only recorded at Fz and ERP as only recorded at Pz since it would be practical to attach only one electrode to the scalp, and those are the location-feature combinations that we a priori expect to produce the clearest results. Effects of workload on the alpha band are particularly expected around Pz (for effortful and attentive processing alpha reduction is observed at parietal regions—(Klimesch et al., 2000; Keil et al., 2006). Effects of workload on the theta band are particularly expected around frontal electrode locations such as Fz (e.g., Miyata et al., 1990; Raghavachari et al., 2001; Jensen and Tesche, 2002; Esposito et al., 2009). The P300 is expected to be most clearly visible at Pz (e.g., Ravden and Polich, 1999; Srinivasan, 2007). Since a priori Pz seems to be the most informative electrode, we also looked at EEG data in general coming only from this electrode.

Skin conductance level was determined by averaging skin conductance over each block. Inspection of the raw data showed that frequently, skin conductance peaks around the onset of a block (i.e., after rating subjective workload of the previous block) after which skin conductance rapidly decreases and remains around the same level. This led us to also use minimum skin conductance of each block as a feature.

As a measure of heart rate, we determined the mean RRI for each block. RRI is the interval between successive heart beats or more precisely, the interval between subsequent R-peaks in the ECG. Three measures of heart rate variability were computed. The root mean squared successive difference (RMSSD: Goedhart et al., 2007) between the RRIs reflects high frequency heart rate variability. High-frequency heart rate variability was also computed as the power in the high frequency range (0.15–0.5 Hz) of the RRI over time using Welch's method applied after spline interpolation; similarly, for mid-frequency heart rate variability the power in the frequency range of 0.07–0.15 Hz was used.

The respiration signal was filtered using a running Gaussian blurring window (with a kernel width of 0.39 s). Subsequently peaks and throughs were detected using the derivative of the signal. Breathing frequency was defined as the mean time interval between the peaks. Modulation depth was defined as the average difference between peak and through.

Pupil size as determined by the Tobii Eyetracker and the ClearView algorithms was averaged for each block. When the eyetracker did not detect the pupil for both eyes for minimally two successive frames (i.e., 33 ms) and maximally 25 successive frames (416 ms), this was considered to be a blink. For each blink, blink duration was determined. Blink rate is the average number of blinks per minute.

The feature “time” was operationalized as the mid-time of the corresponding data segment in seconds from the start of the experiment, discarding breaks and periods in between blocks in which the RSME was registered. For instance, the mid-time of the first 2-min workload block is 60 s and that of the second is 180 s.

Physiological features that were considered with respect to their capacity to estimate workload in this study are summarized in Table 1. This table also indicates the length of the corresponding feature vector (“Dimension”), as well as the single sensors and the sensor groups that the features belong to. For examining the usability of different variables for assessing workload, we follow the list of features as summed up in Table 1. Only for EEG, features can consist of multiple values (dimension larger than 1). The rationale behind this is that the mentioned features are the smallest possible pieces of information that are expected to reflect workload. For examining EEG sensors “all electrodes,” “Pz,” and “Fz,” we only include features reflecting both ERP and spectral properties of the EEG signal as printed in italics. The EEG sensor group only includes ERP and spectral power features of “All electrodes.”

**Table 1:** **Examined (neuro)physiological features, sensors and sensor groups**.

**Sensor group**	**Sensor**	**Feature**	**Dimension**
EEG	*All electrodes*	ERP (0–1 s from stimulus onset)	707
		Spectral power (2–20 Hz)	259
		Alpha power (8–13 Hz)	7
		Theta power (4–8 Hz)	7
		*ERP + Spectral power features*	966
	Pz (single electrode)	ERP (0–1 s from stimulus onset)	101
		Alpha power (8–13 Hz)	1
		*ERP + Spectral power features*	138
	Fz (single electrode)	Theta power (4–8 Hz)	1
Physiology	Skin conductance electrodes	Mean skin conductance level	1
		Minimum skin conductance level	1
	Respiration belt	Respiration frequency	1
		Respiration modulation depth	1
	ECG electrodes	HR (heart rate—RRI)	1
		RMSSD	1
		Mid frequency HRV	1
		High frequency HRV	1
Eye	Eye camera	Pupil size	1
		Blink rate	1
		Blink duration	1

For EEG, the sensors “All electrodes,” “Pz” and “Fz” are examined using the features as defined by ERP and spectral power (printed in italics). The EEG sensor group only includes ERP and spectral power features of “All electrodes.”

### Classification analysis

The first three sessions, each containing two blocks of each n-back condition, were used to train the model parameters to individual participants. The last session was used to evaluate the model's classification accuracy. This simulates estimating workload online, using model parameters that are adjusted to the individual participant in a training phase. As a default, the classification models were trained and applied to distinguish between 0- and 2-back blocks, each containing 2 min of data or 48 trials (letters). Average classification performance (fraction correct in the last session) over all participants was used as measure of model performance.

Feature vectors were constructed for each of the data segments. For instance, the feature vectors used for the model that includes all spectral power values over 120 s blocks of data contains 259 features (power at 37 frequencies × 7 channels, see Table 1 second row) × 16 blocks (4 sessions × 4 blocks). The data from the first 3 sessions was used to train a classifier model for each individual participant. The features were standardized to have mean 0 and standard deviation 1 on the basis of data from the training set. The same standardization transformation was applied to the test data (the data of the 4th session). After training the model using the training data (12 blocks of 259 features in the example above), the classification was applied to the test data and the performance score of each of the individual models was determined. Finally, overall performance is calculated by taking the average score over all individual models.

Classification accuracy was determined for a range of models differing in the (types of) features that were included in the model, differing in the type of classifier and differing in the fusion rule that was used. Classification was performed using the Donders machine learning toolbox (DMLT) developed by van Gerven et al. (2013). Two types of classifiers were used. We used a linear Support Vector Machine as representing a more standard model and, in order to obtain confidence measures that can be used to fuse information, we used an elastic net model with logistic regression (Friedman et al., 2010).

For combining information across sensor groups, both fusion at feature level and fusion at the decision level were investigated. In the first (default) case the concatenated feature vector containing all features was used as the input to a single model. In the latter case, the final decision was based on the average of the probability estimates supplied by the logistic regression from the different elastic net models, each based on the individual features (one model output for each feature). For instance, if estimated probabilities on high workload would be based on mean heart rate, mean skin conductance and blink rate with model output probabilities of *p*1 = 0.2, *p*2 = 0.6, *p*3 = 0.5, the average probability of the combination model is 0.43. Thus, these data would be assessed to reflect low workload.

### Statistical analysis

**Figure 1:**
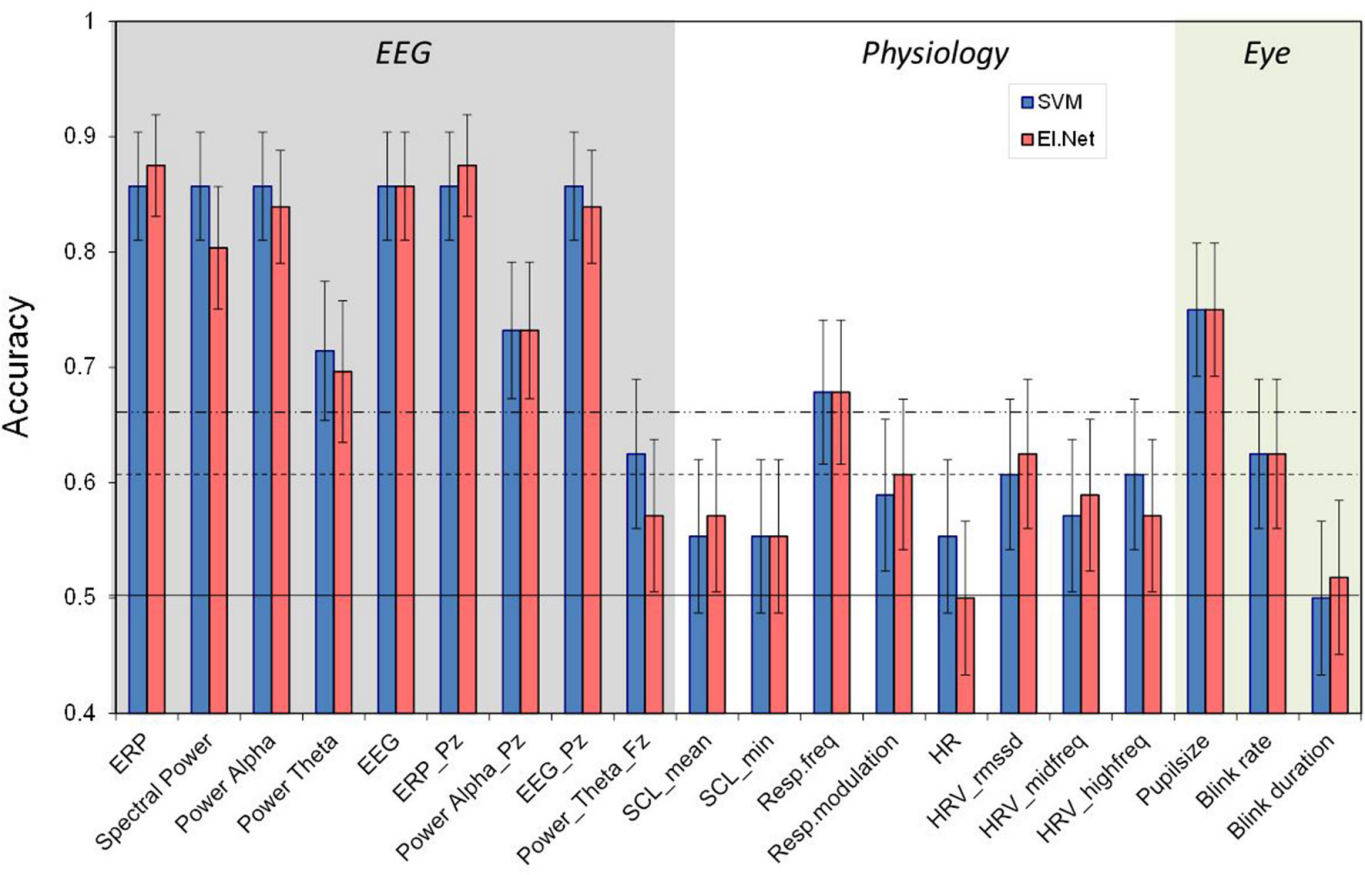
**Classification performance (2- vs. 0-back, 120 s) for separate features as resulting from SVM and elastic net classification models**. The horizontal lines indicate chance level (**bottom**), 0.05 significance level (**middle**) and 0.01 significance level (**top**). Different shades in the background indicate the three different sensor groups EEG, Physiology and Eye. Error bars indicate estimates of the variance (s.e.m.s) based on the binomial distribution.

We used one-tailed binomial tests to determine whether classification accuracy was significantly higher than chance, which works as follows. In the default situation of classifying the 2-min high and low workload blocks (2- vs. 0-back), classification accuracy per participant could only take values of 0, 0.25, 0.50, 0.75, and 1 (correct classification of 0–4 blocks in the last session). To test whether on the whole, classification performance is above chance, we compute the averaged score over all 14 individual models as a measure of performance. This average score can take on values between 0/56, 1/56, 2/56,… 1 (resolution of 1/56 where 56 is 4 possible scores higher than zero^*^14 participants). To determine whether this average score is significantly higher than chance we calculate the chance that a this score or higher is obtained when using a random classification model (i.e., with a probability of classifying a block as one or the other with a probability of 0.5). In this way, one can determine that the chance of obtaining a value of 0.61 or higher given a probability of 50% is equal to 0.05 (the level corresponding to *p* = 0.05 in e.g., Figure 1). We also calculated Bonferroni corrected levels per comparison/figure, and found that when the *p* = 0.05 significance level is corrected for multiple testing (using Bonferroni) this level goes up to the same level as the uncorrected *p* = 0.01 level. This means that the conditions that reach an uncorrected level of *p* = 0.01 maintain significance after Bonferroni correction (at *p* = 0.05).

Pairwise comparison tests were used to determine whether two accuracies were significantly different from each other. To indicate the level of significance chance and alpha levels are shown in the various figures. The figures also include estimates of the standard error in the fractions correct based on a binomial distribution. We did not correct for multiple testing which means that estimates of significance levels are on the low side.

Since the results suggested that EEG models might perform at ceiling level and that we could get a higher benefit of combining variables for a more difficult case where workload assessment did not reach ceiling, we also analyzed performance for classifying smaller workload differences (2- vs. 1-back and 1- vs. 0-back) and for classifying 30 rather than 120 s segments of data. In the latter case two of the participants' data were incomplete due to segments without blinks resulting in undefined blink duration and were discarded (leaving 12 participants). Using parts of blocks rather than complete blocks resulted in having 16 data sets (each 30 s long) per participant available to test the trained classification model rather than 4 (each 2 min long).

## Results

Task difficulty and subjective effort (workload) were successfully manipulated as indicated by the expected effects of n-back level on performance and subjective ratings (Brouwer et al., 2012). The different n-back levels resulted in the expected differences in performance for the 14 subjects with decreasing fraction correct (0.96, 0.94, and 0.90 for the 0-back, 1-back, and 2-back conditions) and increasing response times (560, 616, and 730 ms respectively). Perceived mental effort as measured by RSME increased with n-back level (31, 39, and 55 for the 0-back, 1-back, and 2-back conditions respectively).

### Single variables

Figure 1 shows the performance of models that include a single variable or feature (as defined in the second column of Table 1), separately for the SVM and elastic net classification approaches (see below “Classification Approach”). The horizontal lines indicate chance level, and levels corresponding to a significant difference from chance, for *p* = 0.05 and *p* = 0.01. In general, performance of models based on EEG variables is much better than models based on the other (single) variables.

ERP and spectral power (when using SVM) lead to approximately the same high classification performance of over 0.85 as using all EEG features. Moreover, when reducing information from using EEG or ERP as recorded at all electrodes to only Pz classification performance remains at the same level. Also, when instead of using all frequency bands only alpha is used, performance does not deteriorate. Using only Pz for alpha alone does reduce performance relative to using all electrodes (*p* < 0.05). A model based on the theta band alone does not perform as well as using all frequencies (*p* < 0.05), indicating that the theta band is less informative in our case (in correspondence with our previous findings, see Brouwer et al., 2012).

Models based on the physiological variables perform relatively poorly with respiration frequency being the only feature that reaches the 0.01 significance level with an accuracy of 0.68. High frequency HRV (as defined by RMSSD and spectrally defined) is the only other physiological feature that, depending on the classification approach, just reaches significance (*p* < 0.05).

In comparison, models based on eye measures show relatively good performance with a classification accuracy of 0.75 for pupil size. Blink rate significantly performs above chance as well but blink duration does not.

### Single sensors

**Figure 2:**
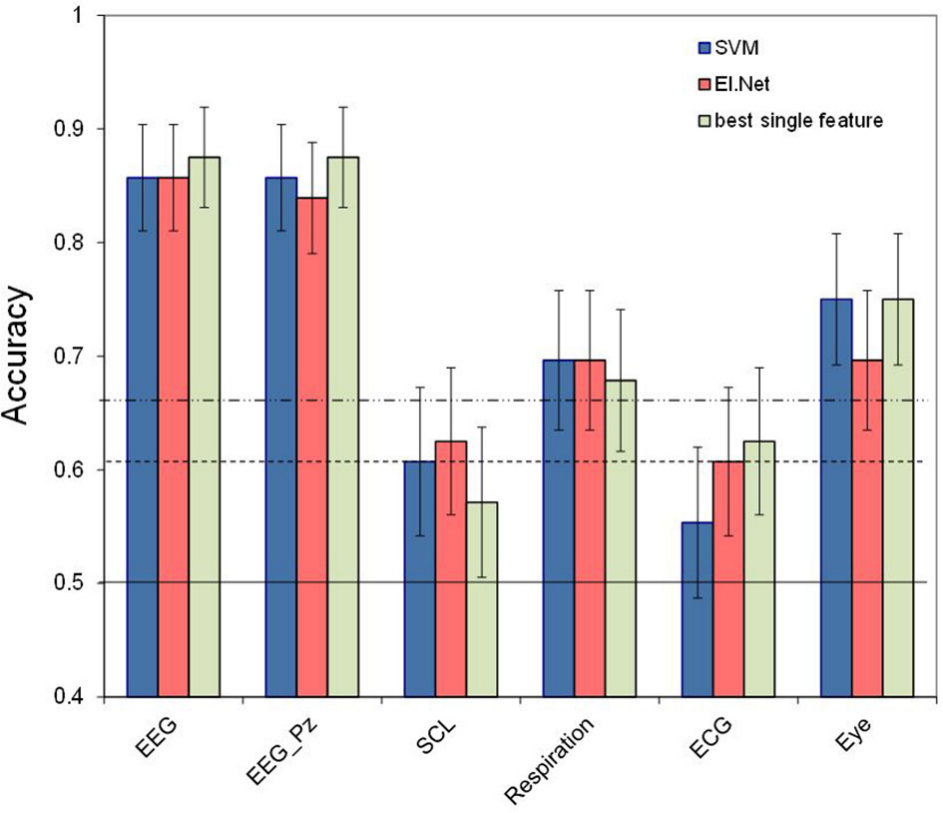
**Classification performance (2- vs. 0-back, 120 s) for separate sensors**. Conventions as in Figure 1. For comparison, performance of the best performing feature for each of sensor is depicted.

Figure 2 shows the performance of the “single sensor” models, i.e., the models that include all features belonging to a certain sensor. Also shown is the performance of the best performing single variable model for each sensor type (in which ERP and spectral power are regarded as the corresponding single features for EEG and EEG_Pz. Skin conductance reaches the significance level when features are combined using the elastic net model, while it remains below significance level for each individual feature. However, and as hypothesized, performance of models using combinations of features from a single sensor do not perform significantly better compared to using only the best performing single feature for any of the sensors. Again, the EEG model shows the best performance (with an accuracy of 0.86 for both classification approaches). Second best is the performance of the models based on eye measures (accuracy of 0.75 for SVM). Also the model based on respiration reaches a relatively high performance level (accuracy of 0.70 for both classification approaches). Models based on skin conductance (accuracy of 0.63 for elastic net) and ECG (accuracy of 0.61 for elastic net) show relatively poor performance, just reaching a level that is significantly higher than chance (*p* < 0.05). Pairwise comparison tests (using the SVM-data) show that the EEG models are significantly different from the other models (*p* < 0.01), and that performance of the eye model is significantly better than that of the skin conductance and ECG models (*p* < 0.05).

### Combinations across sensor groups

**Figure 3:**
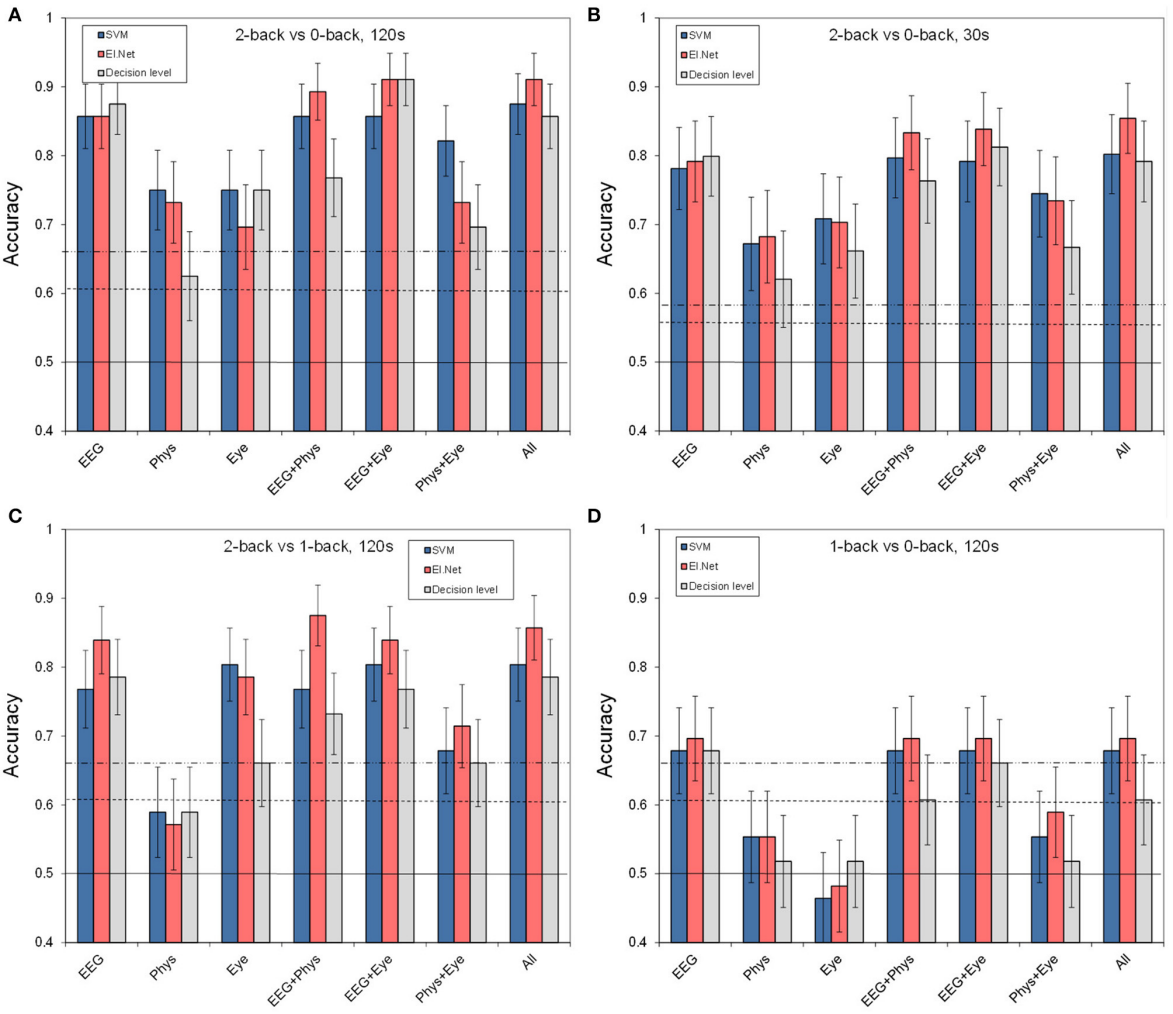
**Classification performance for separate and combined sensor groups for SVM, elastic net and a model that combines the outputs from different single feature models (“decision level”). (A)** performance in the default condition (2- vs. 0-back, 120 s of data, **(B)** for comparing 2- vs. 0-back over 30 s of data, **(C)** for comparing 2- vs. 1-back (120 s of data), **(D)** for comparing 1- vs. 0-back (120 s of data).

Figure 3 shows the results for different (combinations of) sensor groups of SVM and elastic net, as well as the outcome of combining the outputs of different elastic net models (“decision level”). Shown are the results for the default case of classifying 2 vs. 0-back over 2 min. data segments (a) as well as for more difficult cases: using 30 s data segments (b), or classifying 2 vs. 1-back (c), or 1 vs. 0-back (d). Comparing performance in the default case (Figure 3A) with that of separate sensors (Figure 2) shows that for Physiology, combining the three sensors leads to a (non-significant) increase in performance (accuracy of 0.75 for SVM, compared to 0.70 for the best performing single physiological sensor respiration). Models that include EEG perform significantly better than physiology and eye models (SVM, pairwise comparisons, *p* < 0.05). Adding sensor groups to the already well performing EEG improves classification accuracy by 3–5% (for adding Physiology or Eye variables with elastic net). For SVM, the combination of physiology and eye measures tends to improve performance relative to either one alone by 7% as well. However, all of these improvements do not reach statistical significance. Using the assumption of a binomial distribution, significance (*p* < 0.05) is reached for differences of around 10%.

We may not observe a larger, statistically significant improvement of combining EEG with physiology (as we had hypothesized) because EEG alone is already performing very well. Therefore, we performed the same analyses on more challenging classification tasks, namely classification of shorter time segments (30 s rather than 120 s) and classification of more similar workload levels (2- vs. 1-back and 1-back vs. 0-back). In addition, this gives us an impression of how much lower classification performance is under these circumstances. Shorter time segments are expected to be more difficult to classify because the extraction information will be less reliable. This is especially obvious for some of the non-EEG measures (e.g., for high frequency heart rate variability minimum durations of 1 min are advised: Task Force of the European Society of Cardiology the North American Society of Pacing Electrophysiology, 1996; Berntson et al., 1997).

Figure 3B shows the performance of the models for classifying data segments of 30 s instead of 120 s. Note that this includes data from 12 instead of 14 participants (see Materials and Methods). The thresholds for significance decrease in this case since the test set contains 16 samples instead of 4. As expected, performance for classifying 30 s segments is lower than for classifying 120 s segments, with a performance that is (on average) 8% (SVM) and 5% (elastic net) lower. Still, performance for models that include EEG is around 0.8 or higher for the elastic net model. The pattern of results is highly similar to that for classifying 120 s segments—thus, there does not seem to be a larger benefit of sensor group combination than in the previous case, suggesting that the lack of improvement of adding non-EEG variables to the EEG model is not due to a ceiling effect.

Figure 3C shows classification performance for classifying 2-back vs. 1-back using 2-min blocks. Figure 3D shows 1-back vs. 0-back classification performance. As expected, performance for discriminating smaller differences in workload is lower than for discriminating 2-back vs. 0-back. Performance is on average 10% (SVM) and 6% (elastic net) lower for 2 vs. 1-back, with performance around 0.85 for elastic net models including EEG. It is 22% (SVM) and 18% (elastic net) lower for 1 vs. 0-back, with performance just below 0.70 for elastic net models including EEG. This suggests a larger increase in workload from 1-back to 2-back than from 0-back to 1-back in accordance with earlier findings (Brouwer et al., 2012, 2014). Again, models that include EEG variables show the best performance. Performance of classification models based on Physiology is not significantly different from chance for both small workload differences, and the Eye based model drops to chance level when distinguishing 1- from 0-back.

Also included in Figure 3 is the performance of the “decision level” model that combines the output of different elastic net models (based on single features). We did not find any significant differences between performance of the elastic net models that use the two types of fusion; trends indicate an advantage of fusion at the feature level compared to fusion at the decision level.

**Figure 4:**
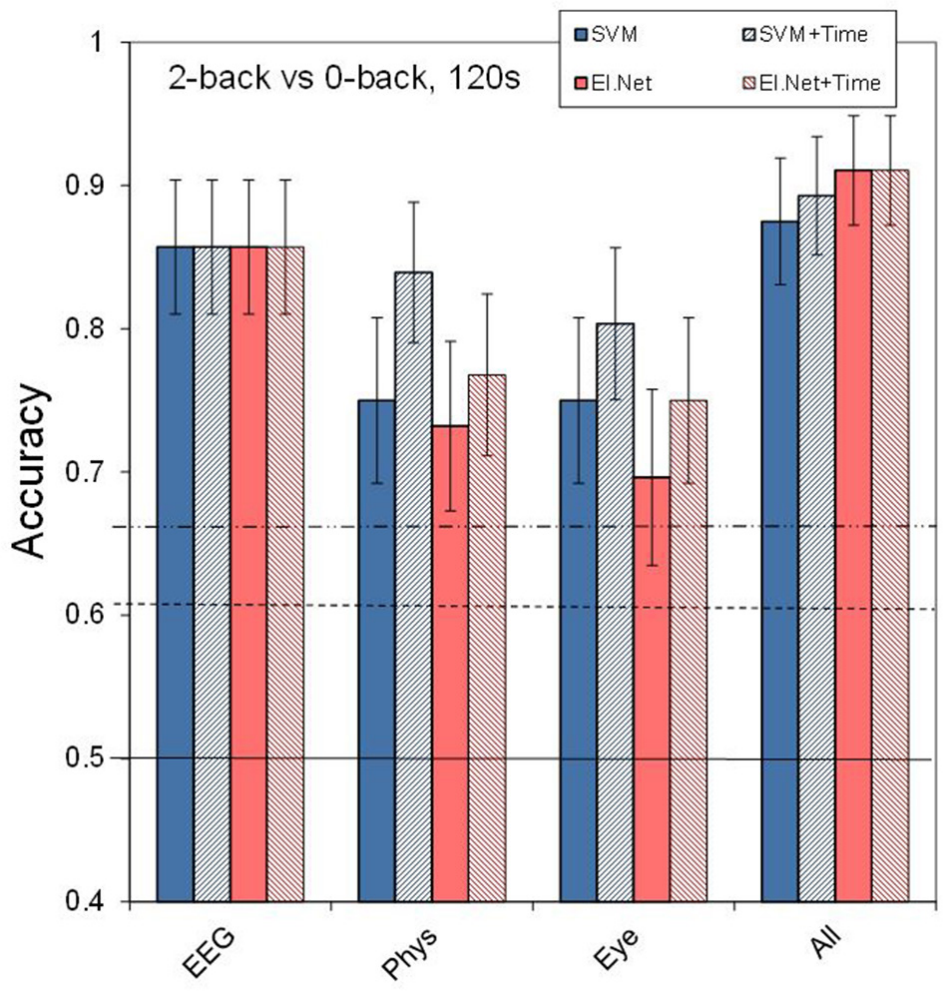
**Classification performance in the default condition (2- vs. 0-back, 120 s) for the different sensor types and using all available input**. The striped bars show the effect of adding the time feature. Conventions as in Figure 1.

Figure 4 shows the effect of adding the feature time (i.e., the time of measurement, since the start of the experiment) to the model input for the default case (2- vs. 1-back, 120 s of data). Adding time leads to an increase in performance of 9% (significant at *p* < 0.05) and 5% (not significant) for respectively physiological and eye sensor group models (SVM) when classifying 2- vs. 0-back using 120 s of data. For EEG and “All” the inclusion of time information does not improve performance. Further analysis of the data shows that when classification is more difficult due to shorter time intervals or smaller workload differences (see Figures 3B–D), the potentially beneficial effect of including time decreases.

## Conclusion and discussion

### Summary of findings

In this study, we compared how well different physiological variables can be used to assess workload in (simulated) real time for a single individual, when the amount of body movement and visual information are controlled for. We also examined to what extent (different ways of) combining information leads to better classification performance, as well as whether taking time into account improves performance.

Classification models based on data from each of the three sensor groups perform above chance (distinguishing high from low workload) at a 0.01 significance level, where EEG reached around 86% classification accuracy and classification models based on peripheral physiology and eye-related variables reached between 70 and 75% accuracy. As hypothesized, the difference in classification accuracy between models based on EEG variables on the one hand, and models based on peripheral physiology and eye-related variables on the other hand was statistically significant. The best performing single variable was ERP at Pz with 88% accuracy (elastic net). All EEG variables (except power in the theta band measured at Fz) performed well above chance (*p* < 0.01). The only non-EEG variables exceeding the 0.01 chance level were respiration frequency (69% accuracy) and pupil size (75% accuracy).

As hypothesized, combining variables recorded using a single sensor (i.e., only the EEG electrodes, electrode Pz, skin conductance electrodes, respiration belt, ECG electrodes or eye camera) does not significantly improve performance over the best performing single variable from that sensor. Variables from the same sensor are likely highly correlated and, in our experiment, combining them has no added value. For four out of the six sensors the trend was even that performance worsened when combining data.

Combining variables of the three physiological sensors (skin conductance electrodes, respiration belt and ECG electrodes) resulted in a modest, non-significant improvement to around 75% classification accuracy with respect to the best performing single physiological sensor (respiration—around 70%). In contrast to what we expected, combining EEG with another sensor group (physiology and eyes) does not lead to a significant improvement in classification accuracy over EEG alone. Using elastic net, we only found non-significant trends of better performance for EEG combined with eye data (91% accuracy) and EEG combined with physiology (89%) than EEG alone (86% accuracy). Adding physiology to fused EEG and eye data does not further improve, or tend to improve, performance. Analysis of data from shorter time segments or smaller workload differences indicates that the fact that we did not get a stronger, significant improvement of adding physiological or eye data to EEG is not caused by a ceiling effect.

Fusion of variables by concatenating feature vectors could not be improved by fusing variables at the decision level. The latter approach results in a more balanced weighting of the different indicators compensating for the low number of features from physiological and eye-based variables relative to EEG, and could therefore have improved classification. As it turned out, the variables that may have profited of the decision level approach, i.e., physiological and eye data, performed lower than EEG variables. Giving more weight to variables with a lower performance is not necessarily beneficial. In addition, classification models based on concatenation may be making more optimal use of interactions between variables that are missed when fusion occurs at the decision level.

Including the time of measurement relative to the start of the experiment as a parameter leads to statistically significant improvements of up to 9% for physiological variables. Adding time did not significantly improve performance of classifiers based on EEG, eye-related variables or combinations of variables. The latter cannot be attributed to a ceiling effect, as indicated by analysis of data from shorter time segments or smaller workload differences.

### Combination of information

The notion that combining physiological variables that reflect a certain mental state will result in a more accurate assessment of this mental state compared to using these variables on their own seems very sensible and has frequently been suggested in the literature as a potential way to improve mental state assessment. However, we are not aware of studies that tried this and showed a statistically reliable and strong improvement. A few studies explicitly mention that combination of physiological information did not result in reliable improvement (e.g., Christensen et al., 2012; Coffey et al., 2012; Severens et al., 2013) or only to a modest degree in one of multiple conditions (Brouwer et al., 2012). Other studies report that classification performance of models combining information increases classification accuracy (by a small amount) but do not provide statistical evidence to show that the effect is reliable (e.g., Chanel et al., 2009). We had anticipated that our study could provide clear evidence for the benefit of combination given the nature of workload, which is a mental state that involves multiple processes that are presumably reflected by different types of physiological variables (e.g., cognitive processes by EEG and arousal by peripheral physiological measures). Also, while most studies combine information by fusion at the feature level, we thought that fusion of information at the decision level could have contributed to finding a strong reliable advantage of combining information. However, we did not find significant differences between the two methods, with the overall trend indicating worse rather than better performance for fusion at the decision. The fact that with this study, there is still no evidence of large benefits when combining physiological variables reflecting workload suggests that they are too highly related to gain a benefit of combination. It could be the case that meta-analyses or a study such as ours including more participants would turn non-significant trends of fusion benefits into statistically significant effects. However, if present at all, the effects of data fusion are at least small. Also, it may be the case that for other tasks (perhaps involving more strong emotional processing besides cognitive processes) benefits of feature fusion can be found more easily.

### Workload assessment performance

Classification accuracy for distinguishing 2-min segments of high vs. low workload for a single individual is relatively high (with an average over participants up to 91%), especially when considering the fact that the amount of movements and visual information was the same across workload levels, and that classification simulated a real time situation.

When the duration of to be classified data segments was decreased to 30 s, this resulted in a relatively small decrease in performance, of on average 5% (elastic net) to 8% (SVM). The results further show that discrimination between more subtle differences in workload is possible as well. These results are good news for potential use in applications. Another finding that is useful for practical applications is that performance based on a single Pz-channel was found to be comparable to that of a model using all EEG-channels. This means that in our case a single channel suffices to characterize the EEG.

As reported, we found EEG variables to be most informative when assessing workload. However, we also found pupil size and blink rate to reflect workload. This is interesting given the fact that lighting conditions and visual input were strictly controlled in our experiment. These results thus indicate that not only pupil size but also blink rate is affected by mental workload level apart from visual demands.

### Group vs. individual level

In the present study, we examine how well different variables can be used to assess workload in real time for a single individual by training classification models on different types of information and comparing performance. Sensitivity of physiological variables is often examined using group level analyses (e.g., using repeated measures ANOVAs). However, for various reasons (see Introduction) one cannot draw straightforward conclusions about assessing workload on an individual level from the results on a group level. For instance, in the current study we found that classification models based on heart rate performed badly, while, for basically the same set of data, this variable was found to be among the ones most strongly associated with workload in a repeated measures ANOVA (Brouwer et al., 2014). Brouwer et al. (2014) also found that heart rate strongly decreased over the time course of the experiment. Since we presented workload conditions in 2-min segments equally dispersed over time, even strong time effects are averaged out in repeated measure ANOVAs whereas they could overrule the comparatively small workload effects in classification type of analyses, especially when classification models are trained on data acquired at the start of the experiment and tested on data at the end. Thus, caution should be taken when generalizing results from studies using group level analysis to situations where momentary data of individuals is used.

### Noise and confounds in real life

In real-life, out-of-the-lab situations, the presence of factors like body movement and varying light conditions may act as noise, therewith diminishing the value of certain variables (e.g., pupil size). Also, levels of workload can be confounded with different levels of stimulus processing or motor actions. For example, workload in Air Traffic Control may be confounded by speech, where controllers talking more during high than during low workload situations. Such confounds may affect physiology (e.g., speech affects respiration), resulting in improved classification accuracy. One of the reasons for the fact that we only found a minor improvement in performance by fusing different workload measures may have been that the experiment controlled for many of such confounding factors, thus decreasing the additional benefit of recording various physiological measures. In this way we were better able to determine which factors directly reflect mental workload. However, in practical situations, physiological measures may supply information about the context and contribute to workload assessment in a more indirect way, i.e., via the confounds. In such a case one should determine whether physiological measures are the most convenient measures to supply workload information, or whether task or behavioral measures (such as the detection of speech through audio sensors with respect to our previous ATC example) are more suitable.

### Conflict of interest statement

The authors declare that the research was conducted in the absence of any commercial or financial relationships that could be construed as a potential conflict of interest.

## Footnotes

^1^Note that here “online” and “real time” refer to using information as collected over the last half- or several minutes. Especially for certain non-EEG measures, it is not possible to retrieve reliable information from very short intervals.

^2^A total of 35 participants took part in the original experiment (see also Brouwer et al., 2012, 2014). However, we here only considered participants with complete data sets. We also performed similar analyses for all participants for which a subset of data was available. The results from such partial analyses showed the same patterns as presented here.
